# A Case of MET Exon 14 Skipping Mutation: Positive Lung Adenocarcinoma Complicated by Paraneoplastic Neurological Syndrome

**DOI:** 10.7759/cureus.57658

**Published:** 2024-04-05

**Authors:** Takumi Murakami, Yutaro Otomo, Tatsuya Ito, Sachiko Imai, Takehiko Ohba

**Affiliations:** 1 Respiratory Medicine, Ome Municipal General Hospital, Tokyo, JPN; 2 Thoracic Surgery, Ome Municipal General Hospital, Tokyo, JPN

**Keywords:** met exon 14 skipping mutation, paraneoplastic syndromes, paraneoplastic neurological syndromes, pulmonary adenocarcinoma, non-small cell lung cancer (nsclc)

## Abstract

A 67-year-old man with cervical spondylotic myelopathy undergoing conservative treatment presented with subacute progression of fine motor and ambulatory disturbances, leading to admission at a previous hospital. Pre-cervical laminoplasty chest computed tomography (CT) revealed a tumor in the left upper lobe of the lung, prompting transfer to our institution. Transbronchial biopsy findings were consistent with adenocarcinoma, diagnosed as clinical stage T2bN0M0, Stage IIA. The neurological abnormalities could not be solely attributed to cervical spondylotic myelopathy, leading to a diagnosis of concurrent paraneoplastic neurological syndrome (PNS). During hospitalization, the patient's condition progressed to a state of constant bed rest within two weeks. On the 17th hospital day, a left upper lobectomy was performed, resulting in significant improvement, allowing the patient to ambulate with assistance after two weeks, and transfer to a convalescent rehabilitation hospital on the 58th hospital day. Subsequent cancer multigene panel testing revealed a positive MET exon 14 skipping mutation. Given the absence of reports on this mutation in lung adenocarcinoma associated with PNS, we consider it rare and thus report this case.

## Introduction

Paraneoplastic neurological syndrome (PNS) is a relatively rare condition among cancer patients, characterized by neuromuscular disorders that present neurological symptoms due to immunological mechanisms, rather than through metastasis or direct invasion [1,2]. Small cell lung cancer (SCLC) is a typical example, while non-small cell lung cancer (NSCLC) is rare [3]. We encountered a patient with lung adenocarcinoma and concurrent PNS, who showed significant improvement after surgical treatment. Cancer multigene panel testing on the postoperative specimen revealed a positive MET exon 14 skipping mutation. Given the lack of reports on lung adenocarcinoma with this mutation associated with PNS, we considered this a rare case and herein report it.

## Case presentation

A 67-year-old man with hypertension and dyslipidemia noticed abnormal sensations in his left upper limb two months before referral to our institution, for which he consulted a previous hospital. He was diagnosed with cervical spondylotic myelopathy and managed conservatively. One month before referral to our hospital, he began experiencing abnormal sensations and weakness in his right upper limb, which worsened over time. Although cervical laminoplasty was planned, a chest computed tomography (CT) before surgery revealed a tumor shadow in the left upper lobe of the lung. Therefore, he was transferred to our hospital the following day for further evaluation.

The patient had a smoking history with a Brinkman index of 920 and drank alcohol only occasionally.

The patient was alert and showed no abnormalities in cranial nerves. Bilateral muscle weakness, predominant on the distal left side, and impaired fine motor skills were observed in the upper limbs, while bilateral muscle weakness, with a proximal left-side predominance, was noted in the lower limbs. Sensory loss in touch was identified in the left C5 to C7 dermatomes and the left L5 dermatome, with pain sensitivity reduction in the left C5 to C7 dermatomes and a marked decrease in vibration sense at the right ulnar head. Deep tendon reflexes were generally hyperactive, though diminished in specific areas (left brachioradialis reflex, right patellar reflex).

**Figure 1:**
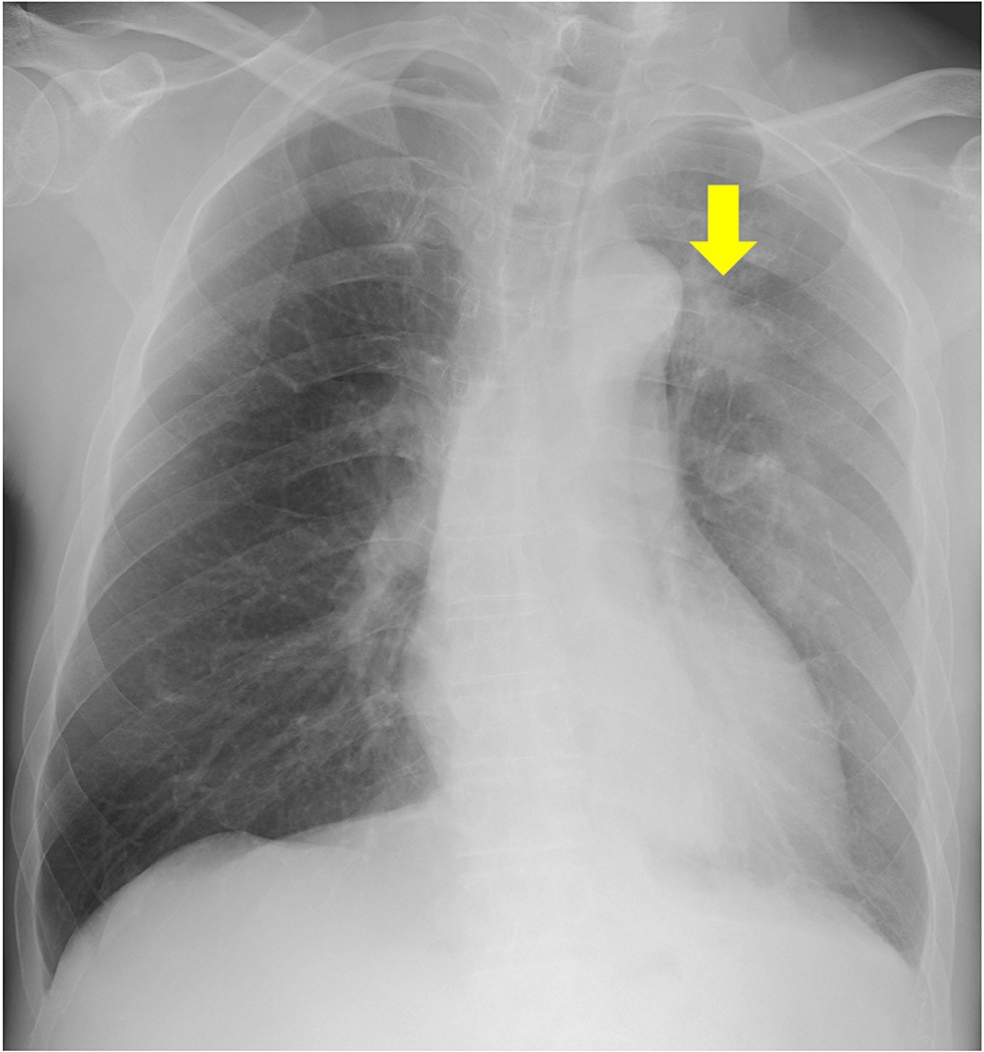
Chest radiography on admission shows a mass shadow in the upper left lung field (arrow).

**Figure 2:**
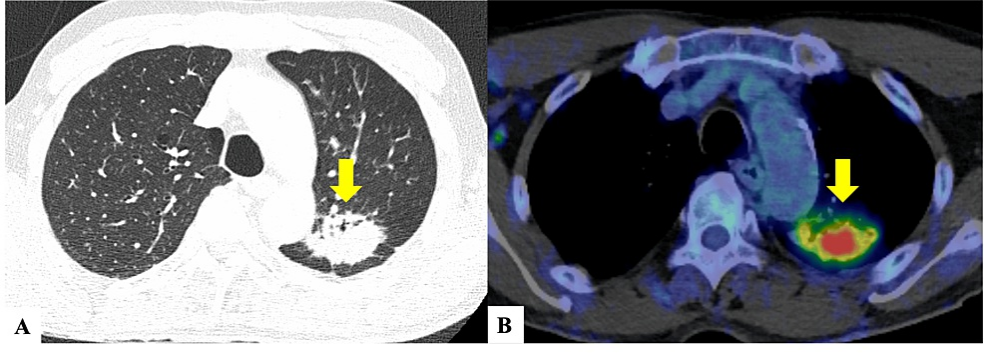
CT scans (A) Chest plain CT shows an irregular mass shadow measuring 47 mm in the upper lobe of the left lung (arrow). (B) On FDG-PET/CT, high FDG uptake with an SUVmax of 9.8 in the tumor of the left upper lobe (arrow). FDG: fludeoxyglucose-18; PET: positron emission tomography

Chest X-ray revealed a mass shadow in the left upper lung field (Figure 1) and contrast-enhanced CT showed an irregular mass measuring 47 mm in the upper lobe of the left lung (Figure 2A). There was no enlargement of the mediastinal hilar lymph nodes. Brain contrast-enhanced magnetic resonance imaging (MRI) did not identify any evidence of brain metastases or meningeal carcinomatosis. F-fluorodeoxyglucose positron emission tomography/computed tomography (FDG-PET/CT) identified high FDG uptake with an SUVmax of 9.8 in the tumor of the left upper lobe (Figure 2B).

**Figure 3:**
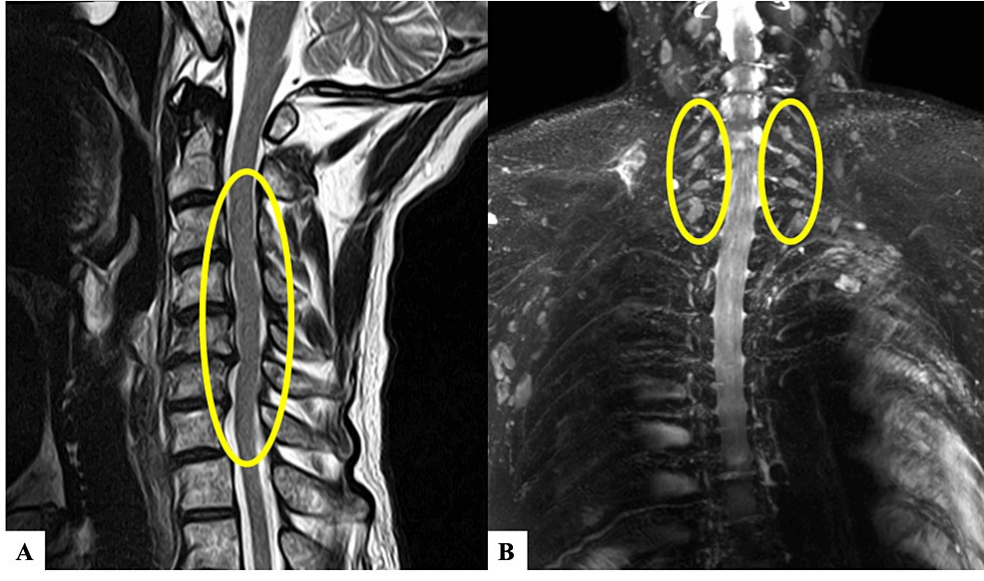
MRI scans (A) Cervical spine plain MRI reveals spinal canal stenosis from C3 to C7, with no changes in signal intensity within the spinal cord (circle). (B) Cervicothoracic spine contrast-enhanced MRI (MR neurography) shows an enlargement of the dorsal root ganglia from C4 to C7 and enhancement with contrast medium (circle).

Cervical spine plain MRI demonstrated spinal canal stenosis from C3 to C7 without any changes in signal intensity within the spinal cord (Figure 3A). Cervicothoracic spine contrast-enhanced MRI showed an enlargement of the dorsal root ganglia from C4 to C7 and enhancement with contrast medium (Figure 3B).

Analysis of the cerebrospinal fluid revealed findings of albuminocytologic dissociation (total protein: 168.3 mg/dL, white blood cells (WBC) 3/μL [all mononuclear cells]), but oligoclonal IgG bands were negative. Additionally, cytology and culture were negative.

Blood tests showed elevated levels of the following: WBC at 10,960 /μL, lactate dehydrogenase (LDH) at 242 IU/L, C-reactive protein (CRP) at 5.43 mg/dL, and carcinoembryonic antigen (CEA) at 12.9 ng/mL. Vitamin B1 (38.8 ng/mL), vitamin B12 (632 pg/mL), and folic acid (4.3 ng/mL) were all within normal ranges, indicating no vitamin deficiency. Autoantibodies associated with connective tissue diseases, including antinuclear antibodies (ANA), anti-SS-A antibodies, anti-neutrophil cytoplasmic antibodies (ANCA), anti-aminoacyl-tRNA synthetase (ARS) antibodies, and anti-transcription intermediary factor 1 (TIF1)-γ antibodies, were all negative. A panel of anti-neuronal antibodies including anti-Ri, anti-Yo, anti-Hu, anti-amphiphysin, anti-CV2, anti-Ma2, anti-Recoverin, anti-SOX1, anti-Titin, anti-Zic4, anti-GAD65, and anti-Tr antibodies were tested and all found to be negative. Urinalysis showed no abnormalities.

On the third hospital day, a transbronchial biopsy of the tumor in the upper lobe of the left lung was performed, revealing adenocarcinoma predominantly characterized by lepidic growth.

Based on the findings, the diagnosis was adenocarcinoma in the upper lobe of the left lung, clinical stage T2bN0M0, Stage IIA. The subacute progression of neurological symptoms presented as multifocal sensory and motor neuropathy, which could not be accounted for by cervical spondylosis alone. Referring to the diagnostic criteria proposed by Graus et al. [4], the presence of acute/subacute sensory and motor peripheral neuropathy, along with the concurrent malignancy, led to the diagnosis of possible PNS.

**Figure 4:**
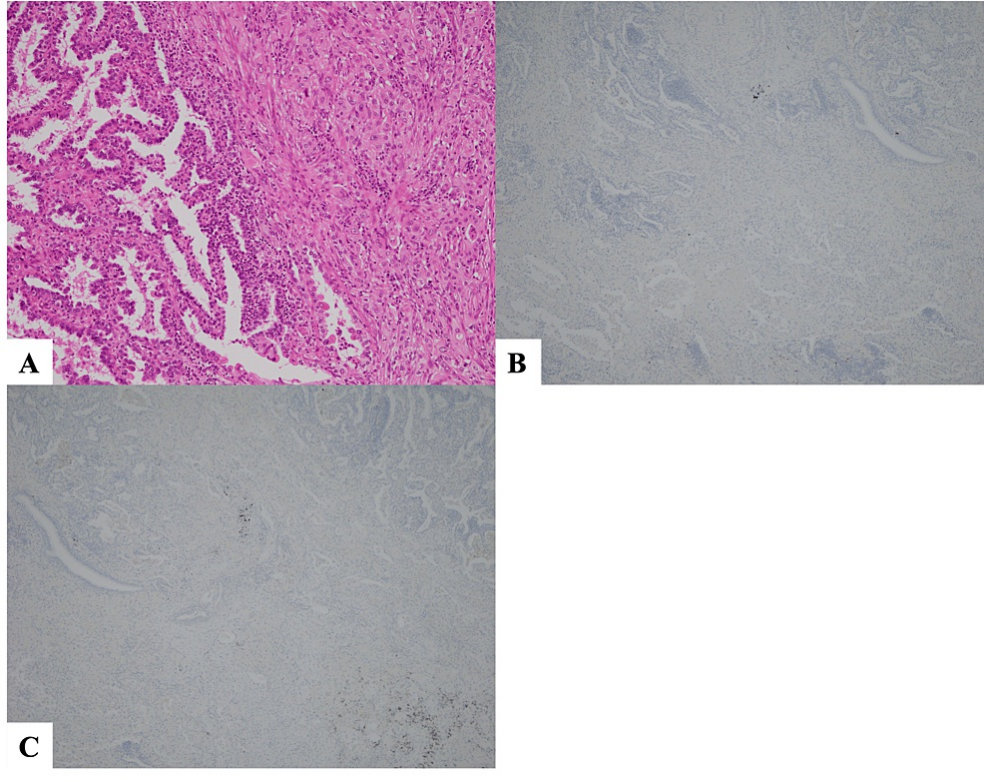
Pathological findings of the surgical specimen. (A) Left side: lepidic adenocarcinoma is observed under HE staining. Right side: tissue composed of somewhat poorly differentiated adenocarcinoma cells is observed (Magnification 100x). (B) Immunostaining for Chromogranin A is negative (Magnification 40x). (C) Immunostaining for synaptophysin is negative (Magnification 40x).

The patient's neurological symptoms were progressive, resulting in an inability to walk and a bedridden state by the 14th hospital day. Given that the lung cancer was at a potentially curative stage (Stage IIA), a thoracoscopic left upper lobectomy and systemic lymph node dissection was performed on the 17th hospital day in hopes of improving the neurological symptoms. Postoperative pathology confirmed adenocarcinoma (70% lepidic adenocarcinoma, 30% acinar adenocarcinoma) under Hematoxylin-Eosin (HE) staining (Figure 4A). Although it contained poorly differentiated components, no cells suggestive of small cell carcinoma were found. Immunohistochemically, even in areas of low differentiation, neuroendocrine markers chromogranin A and synaptophysin were negative (Figures 4B-4C).

Moreover, a cancer multigene panel test conducted on the surgical specimen identified a positive MET exon 14 skipping mutation.

Postoperatively, the neurological symptoms gradually improved, and by the 32nd hospital day, the patient was able to walk with assistance. Sensory deficits in touch, pain, and vibration improved on a weekly basis. The patient was transferred to a convalescent rehabilitation hospital on the 58th hospital day for ongoing rehabilitation.

Currently, the patient is under outpatient follow-up, able to visit the hospital unassisted, without any recurrence of neurological symptoms or lung cancer.

## Discussion

PNS refers to neuromuscular disorders in cancer patients that manifest neurological symptoms not due to metastasis or direct infiltration but arise from immunological mechanisms [1]. They are reported to occur in 0.3% of all malignancies, making them relatively rare conditions [2]. The majority of malignancies associated with PNS are lung cancers, with SCLC being the most common, whereas NSCLCs such as lung adenocarcinoma are rare [3,5]. In this case, given the background of NSCLC, the coexistence of neuroendocrine tumors was considered and assessed; however, both morphologically and immunohistochemically, this hypothesis was dismissed.

In many cases of PNS, the appearance of neurological symptoms and the presence of anti-neuronal antibodies precede the detection of the malignancy. It has been reported that an average period of four months is required from the onset of neurological symptoms to the definitive diagnosis of malignancy [6]. In this case, the interval from the onset of neurological symptoms to the identification of the malignancy was approximately two months, consistent with previous reports.

In 2004, Graus et al. established diagnostic criteria for PNS, classifying them as either definite or possible based on the presence or absence of a malignancy, characteristic neurological symptoms, and the combination of anti-neuronal antibodies [4]. This case was evaluated using the same diagnostic criteria. The findings from cervicothoracic spine contrast-enhanced MRI suggested the coexistence of dorsal root ganglionitis, and cerebrospinal fluid analysis showed albuminocytologic dissociation. However, the neurological findings did not match the selective sensory nerve impairment (sensory neuronopathy) typical of dorsal root ganglionitis [7]. Therefore, it was assessed as an acute/subacute sensorimotor neuropathy under non-classical syndrome, resulting in a diagnosis of possible PNS. Nonetheless, the significant improvement in neurological symptoms following surgical treatment for lung adenocarcinoma led to the final diagnosis of definite PNS. It is worth noting that, although anti-neuronal antibodies were negative in this case, it has been reported that approximately 40% of lung cancers with concurrent PNS are negative for anti-neuronal antibodies [8].

Treatment of PNS often involves the use of immunosuppressive therapies such as steroids, intravenous immunoglobulin therapy, and plasmapheresis based on the pathophysiology. However, these approaches rarely yield effective results [9]. Generally, cancer treatment is considered the first choice [10], and in this case, surgical treatment was promptly administered, resulting in an improvement in neurological symptoms. However, the efficacy of cancer treatment is reported to be around 10% [11], indicating that it is not effective in all cases.

A cancer multigene panel testing performed on the surgical specimen identified a positive MET exon 14 skipping mutation. To our knowledge, there have been no previous reports of the MET exon 14 skipping mutation-positive NSCLC complicated by PNS, making this case the first of its kind. Furthermore, regarding NSCLC with driver mutations associated with PNS, we found only two case reports of epidermal growth factor receptor (EGFR) mutation-positive NSCLC complicated by PNS, indicating that it is an extremely rare condition [12,13]. The relationship between driver mutations, including MET exon 14 skipping mutations, and PNS has not been established, necessitating further accumulation of cases and molecular biological investigations.

## Conclusions

We encountered a case of MET exon 14 skipping mutation-positive lung adenocarcinoma complicated by PNS. In patients with cancer, when progressive neurological symptoms that cannot be explained by a single pathology are observed, PNS should be actively considered in the differential diagnosis. Given the improvement observed following surgical treatment in this case, we believe that aggressive tumor treatment can contribute to the improvement of patient prognosis in cases of PNS. Moreover, the relationship between driver mutations, including the MET exon 14 skipping mutation identified in this case, and PNS has not been clarified, underscoring the need for further case accumulation.
